# Power law relations in earthquakes from microscopic to macroscopic scales

**DOI:** 10.1038/s41598-019-46864-8

**Published:** 2019-07-24

**Authors:** Fanzhen Meng, Louis Ngai Yuen Wong, Hui Zhou

**Affiliations:** 1 Department of Earth Sciences, The University of Hong Kong, Pokfulam, Hong Kong China; 2 College of Science, Qingdao University of Technology, Qingdao, Shandong 266033 China; 3 HKU Shenzhen Institute of Research and Innovation (HKU-SIRI), Shenzhen, Guangdong 518057 China; 4 State Key Laboratory of Geomechanics and Geotechnical Engineering, Institute of Rock and Soil Mechanics, Chinese Academy of Sciences, Wuhan, Hubei 430071 China

**Keywords:** Natural hazards, Natural hazards, Seismology, Seismology

## Abstract

Understanding the physics of earthquakes is a crucial step towards improving the prediction accuracy of earthquakes. Scale invariance or fractal features are often reported in earthquakes, such as the size distribution of earthquakes, the spatial distribution of hypocenters, and the frequency of aftershocks. Here we assess whether other key parameters and quantities involved in earthquakes also conform to the power law. By analyzing a large amount of data collected from the laboratory experiments and field monitoring of earthquakes, we find that the crack density on the two sides of small scale fracture or large scale fault decreases with increasing distance following the power law, and the crack number-crack length distribution is also scale invariant like natural faults. Besides, the earthquake b-value is found to decrease with increasing stress in a power law in the brittle regime of the Earth’s crust. The friction coefficient for dry fault and gouges or for partially saturated gouges decreases with the increasing effective normal stress in a power law. The stress dependency of b-value and friction coefficient is dictated by different mechanisms. Our findings will advance the understanding of earthquake physics, and will enable us to better model, predict and conduct hazard assessment of earthquakes.

## Introduction

Earthquake is one of the most catastrophic natural disasters in the world. However, the accurate prediction of the occurrence time and location of earthquakes still remains elusive^1^, despite great efforts already devoted to improving techniques to monitor and analyze earthquakes in the last few decades. The extent to which earthquake hazards can accurately be predicted will ultimately depend on how well the underlying physical conditions and processes are understood^1,2^. Both laboratory testing on small scale rock specimens under controlled conditions^3–6^ and field seismic monitoring^1,7,8^ are important means to throw light on the physics and prediction of earthquakes.

Many man-made and natural phenomena are believed to follow power-law distributions^9,10^. In earthquake science, Ishimoto-Iida law^11^ may be the earliest power law describing the frequency distribution of the maximum amplitude of seismograms in the form of $n(a)=K{a}^{-m}$, in which *a* is the amplitude, *n(a)* is the number of events whose amplitude is *a*, and *m* and *K* are constants. The most well-known power law is the Gutenberg-Richter (G-R) law, which describes the earthquake frequency-magnitude distribution^12^:

$${\mathrm{log}}_{10}N=a-bM,$$

where *N* is the number of earthquakes larger than magnitude *M*, parameter *a* describes the total number of earthquakes, and *b* refers to as *b*-value. The latter gives insights on the relative scaling of large versus small earthquakes^13^, and it was often used to infer tectonic stress^14,15^, predict impending large earthquakes^16–19^ and aid seismic hazard assessments^20,21^. In the laboratory rock fracture test and fault shear test, the size distribution of the acoustic emission (AE) was found to obey the same G-R law^4–6^. Besides, the temporal decay of aftershock rate following a main shock can also be described by another well-known power law aside from G-R law for the frequency and magnitude: the Omori-Utsu law^8,22^:

$${\rm{\Lambda }}(t)=k{(t+c)}^{-p},$$

where *t* is the time from the main shock, ${\rm{\Lambda }}(t)$ is the aftershock rate at time *t*, *k* is the productivity of the aftershock, *p* is the power-law exponent. In addition to the above famous power laws in earthquakes, the spatial distribution of hypocenters^23,24^, the roughness of fault surface and profile length^25,26^, etc, are also found to follow the power law.

Identifying the power-law distribution of data can indicate the presence of unusual underlying or endogenous processes. Knowing whether a quantity follows a power law provides important theoretical clues about the underlying generative mechanisms and facilitates statistical extrapolations about the likelihood of very large events^10^. In earthquake science, exploring whether the power law is a ubiquitous pattern among those microscopic and macroscopic parameters and quantities involved in the earthquake will advance our understanding of earthquakes (physics, modeling, prediction and hazard assessment). In this study, we comprehensively analyze large amounts of data compiled from the laboratory experiments and field monitoring of earthquakes, and examine whether these data conform to the power law distribution.

## Data and Method

The data used in this study is mainly compiled from the literature, and some published and unpublished results by the authors ourselves. We analyze the variation of crack density and crack length around the fault available in both laboratory test results (data from refs^27–31^) and natural fault analysis (data from refs^32,33^). The earthquake b-value in ref.^15^ and the acoustic emission b-value monitored during laboratory triaxial compression test, direct shear test and double direct shear test (data from our unpublished results, refs^5,34,35^ respectively) to simulate earthquakes are used to interpret the stress dependence of b-value. The friction coefficients in refs^36–39^ and our unpublished results respectively obtained by triaxial compression tests, rotary-shear frictional tests and direct shear tests are analyzed to show the variation with stress level. In summary, 20 groups of data are included and analyzed in this study, 5 of which are from the experiments conducted by ourselves, and the other data are collected from the published literatures. When data values of some published plots are not available, we use the *GetData Graph Digitizer* software to digitize the plots and obtain the data values.

## Results

### Crack density distribution away from the fault

Earthquakes are believed to be caused by the sudden slippage along a pre-existing fault or plate interface^3^. Understanding the source processes of earthquakes relies on the understanding of shear fracturing in rocks^40^. A host of studies have been conducted to elucidate details of the microscopic operative mechanism during the faulting process in the laboratory and in the field^27,28,32,41^. The brittle failure of rock in a controlled condition in the laboratory can provide deep insight into the micromechanics of faulting^32,41^.

**Figure 1:**
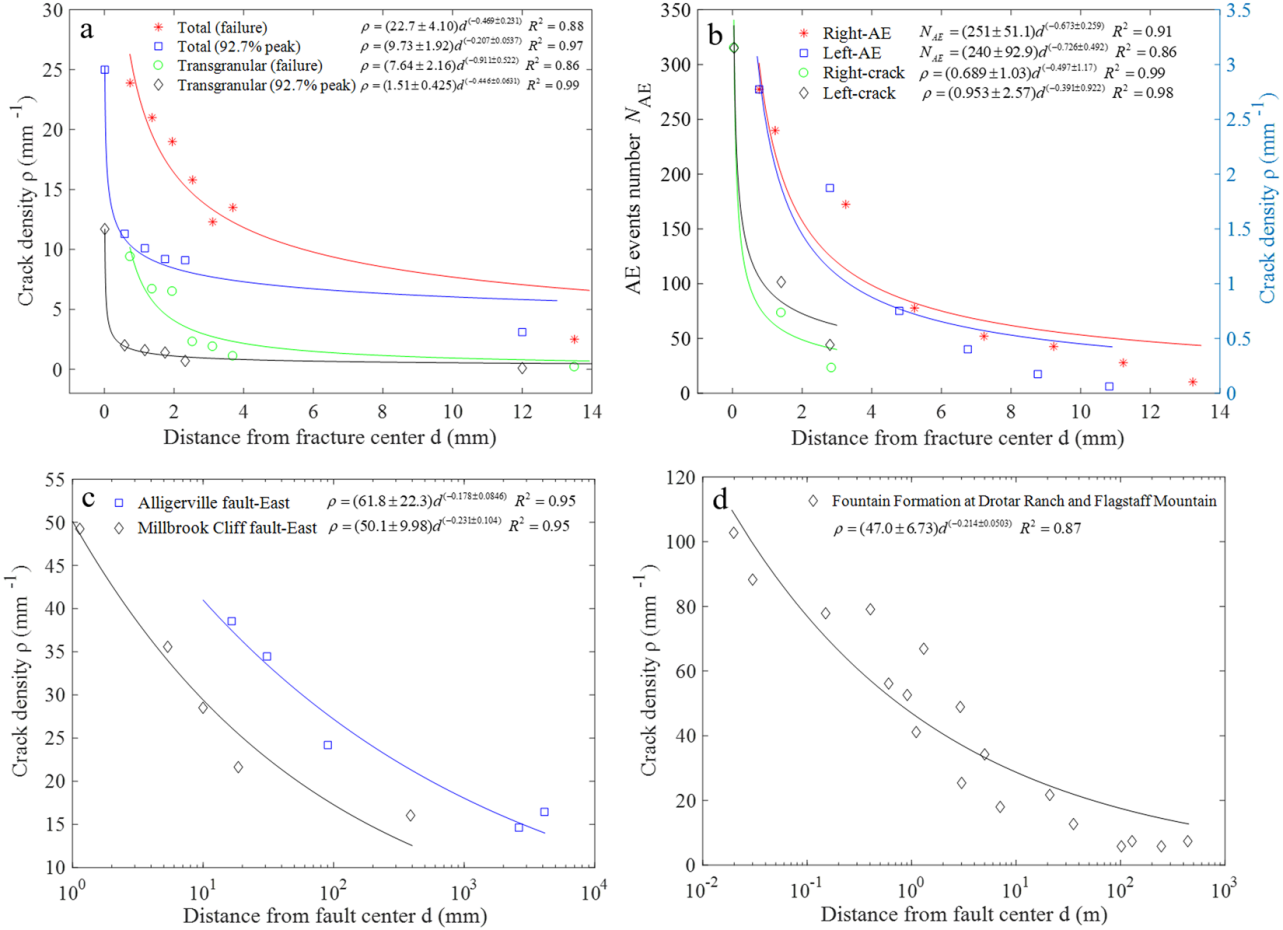
Distribution of crack density away from the fault in laboratory and field scale. (**a**) Total and transgranular cracks density at 92.7% of peak strength and at peak strength for marble specimen in the uniaxial compression test. (**b**) Number of AE events and crack density on the two sides of the shear fracture of Aue granite cores in the asymmetric uniaxial compression test. (**c**) Crack density in the east side of the two natural faults. (**d**) Crack density versus distance to the fault surface for combined data from the Fountain Formation at Drotar Ranch and Flagstaff Mountain, Colorado.

Compression-induced shear fractures in marble specimens containing en-echelon flaws were systematically investigated^28^. Figure 1a shows the crack density counted from the thin sections of those specimens loaded under the uniaxial compression tests up to 92.7% peak strength or after failure. In the former test, the specimen is first loaded to 92.7% of the average peak strength, and then the test is stopped and unloaded. Thin sections are made on this specimen for the subsequent crack density assessment. Both the trans-granular crack density and total crack density (sum of grain boundary cracks and trans-granular cracks) at 92.7% peak strength and after failure decrease non-linearly with the distance away from the medial plane of the shear fracture, the decrease of which can be well fit by the power law (in this study, the fitting relationship between the parameter (*y*) and the variable (*x*) is expressed by $y=(a\pm {c}_{1}){x}^{(-b\pm {c}_{2})}$, *a* and *b* are constants, and *c*_1_ and *c*_2_ indicate the range of 95% confidence interval). Similar test results were also reported in ref.^29^ in the shear fracture tests of granite. In the asymmetric uniaxial compression test performed on Aue granite cores, the microcrack density and the AE events on the two sides perpendicular to the shear fault were investigated^27^ (Fig. 1b, re-plotted based on the data in Fig. 7 of ref.^27^). More but similar test results were shown in ref.^42^. The reported location accuracy of the AE system was 3 mm. The crack density is the average value along the trace of the main fracture in a very small targeted area, and AE event number is the total number at a particular distance from the main fracture. The power laws can be used to describe both the decrease of the crack density and the cumulative AE event with the increase of distance from the center of the fault.

In addition to the laboratory faulting test, field observations on natural faults also indicate that crack density around the fault decreases non-linearly with the increasing distance to the fault^32^. Figure 1c shows the microfracture density-distance plot for Alligerville fault and Millbrook Cliff fault (re-plotted based on the data in Fig. 6 of ref.^32^, and only the data in the east of the faults are plotted due to general data symmetry with the west side). Logarithmic coordinate is used in the horizontal axis due to the very large span. The power law decrease of crack density for natural faults is further supported by the good fit to a power function regression of the combined microfracture data from the Fountain Formation at Drotar Ranch and Flagstaff Mountain in a much larger scale, as shown in Fig. 1d (re-plotted based on the data in Fig. 6c of ref.^33^). Similar to the small scale laboratory fracture test, the crack density in vicinity of a natural fault also decreases non-linearly following the power law.

### Crack length distribution

The crack initiation and propagation play important roles in controlling the brittle failure of rock upon loading^41^. The macro-failure of rock is caused by the coalescence of small scale cracks^41,43^. Understanding the crack length distribution of rock at different stress level is important to throw light on the failure mechanisms, and for the establishment and verification of the micro-scale damage model of rock^4,43,44^.

**Figure 2:**
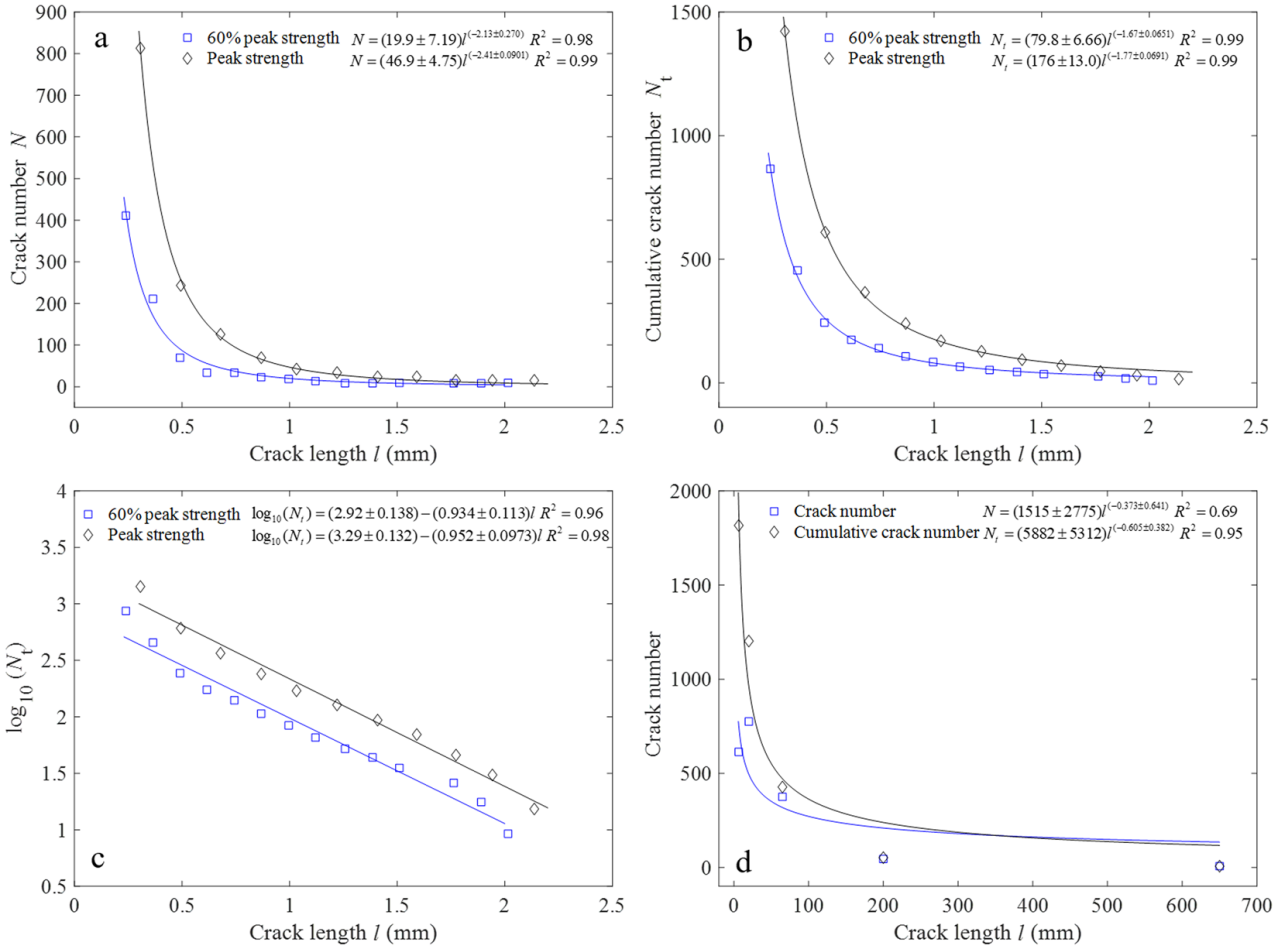
Crack length distribution of granite loaded at different stress level. (**a**) The number of cracks (*N*) with a particular crack length under 5 MPa confining pressure for granite at 60% and 100% of peak strength. (**b**) The cumulative crack number (*N*_*t*_) larger than a particular crack length corresponding to (**a**). (**c**) The fitting linear curve of log_10_(*N*_*t*_) and crack length corresponding to (**b**). (**d**) Crack number-length distribution of Westerly granite at the tip of shear fracture under 50 MPa confining pressure.

Figure 2a,b show the crack length and number relationship of granite under 5 MPa confining pressure, and the figure is re-plotted based on the data in ref.^30^. Figure 2a shows the crack number of a particular length, and Fig. 2b shows the cumulative crack number less than a particular crack length. The two sets of data in each figure are the results of the granite specimens loaded to 60% and 100% (failure) of its peak strength respectively. Figure 2a suggests that the number of long cracks gradually decreases, which can be well fit with the power law relations. The cumulative crack number also decays with power law with increasing crack length, which is very similar to the decay of the number of earthquakes/AE events with increasing magnitude/amplitude. Similar to the approach adopted in the G-R law, by plotting the logarithm of the cumulative crack number in the vertical axis, the new crack frequency-length relation can be determined, as shown in Fig. 2c. The slope of the linear fitting line indicates the proportion of long cracks to short cracks. The comparison of the two slopes of the fitting lines suggests that the difference in the crack length distribution of this granite at different stress level is insignificant.

Figure 2d shows the crack length and number at the tip of a shear fracture of Westerly granite under 50 MPa confining pressure (the data are from ref.^29^, the crack number oriented within 45° and larger than 45° in the original Fig. 3 of ref.^29^ are added up to obtain the total crack number in this study). The cumulative crack number and the crack length are well fit with the power law. The cumulative number decreases sharply at the early stage, while the decrease becomes much slower with increasing crack length, which is similar to the trend in Fig. 2b. However, the number of the shortest crack is fewer than the subsequent longer cracks (denoted by the blue squares in Fig. 2d), which is different from those shown in Fig. 2a. Similar results as shown in Fig. 2d are also reported in ref.^31^, which examined the crack patterns in a small marble plate specimen (20 mm × 5 mm) with real time SEM observation under the uniaxial compression test. As the very small cracks are difficult to be observed, and the resolution varies among observational tools such as optical microscope and SEM, we consider that the different crack patterns as shown in Fig. 2a,d (blue squares) are due to the microcracks of different scale that are considered. The number of the smallest cracks is likely to be the largest, but these cracks cannot be detected due to their small size.

### Stress dependence of b-value

The b-values obtained from both earthquake statistics and laboratory tests of small scale specimens are found to be inversely dependent on differential stresses^5,15^, and the b-value decreases progressively with the increasing stress until failure is reached^4,6,34,35^.

**Figure 3:**
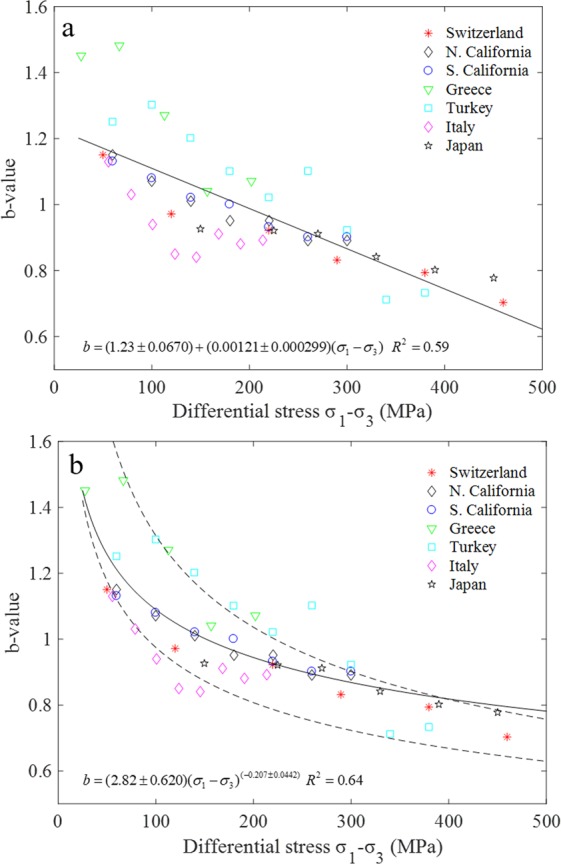
Variation of earthquake b-value with differential stress for a variety of tectonic regions, and the data is replicated from ref.^15^. (**a**) The linear relation between the b-value and differential stress. (**b**) Power law is used to fit the relation between the b-value and differential stress in this study using the whole data set.

Using the b-value data from ref.^14^ and the stresses computed from the stress model (different stress gradients for thrust faulting, normal faulting and strike-slip region), a linear negative relationship between the b-value and the differential stress from various tectonic regions was developed^15^, i.e., $b=1.23\pm 0.06-(0.0012\pm 0.0003)\,({\sigma }_{1}-{\sigma }_{3})$, which is reported to simultaneously explain both the depth and focal mechanism dependence of b-value. The data in Fig. 1 of ref.^15^ is replicated and shown in Fig. 3a, which is then fit by linear fitting. The relation of the b-value and the differential stress can be described by $b=(1.23\pm 0.07)-(0.0012\pm 0.0003)\,({\sigma }_{1}-{\sigma }_{3})$ (for a better comparison, the numbers are rounded to the same number of decimal places as in the originally reported equation, and the fitting formula obtained in the present study can be found in Fig. 3a), which is quite similar to the above one, indicating the accuracy and reliability of the data replication method. However, the correlation coefficient in our study (0.59) is lower than that (0.77) in ref.^15^. A careful re-examination of the data indicates that the linear fitting of the stress and b-value cannot reflect their true relationship, especially in the low (<50 MPa) and high (>300 MPa) stress ranges. The data points in these two ranges deviate from the linear trend. We propose that the power law is more suitable to describe the b-value dependence on the stress (Fig. 3b). In the low stress range, the b-value is large, and also decreases rapidly with the increase of stress. On the other hand within the high stress range (300 to 500 MPa), the influence of stress on the b-value becomes less significant, and the b-value decays at a much lower rate as compared with that before 300 MPa. The correlation coefficient using the power law is 0.64 (Fig. 3b), which is higher than the 0.59 in the linear fitting. Besides, we choose the data points on the up and low boundary of all the data set respectively in Fig. 3a, and fit the two new groups of data with the power law separately, as shown by the two dashed lines in Fig. 3b. The fitting results are strikingly well, and the correlation coefficients are higher than 0.96, further suggesting the superiority of the power law to the linear relationship to describe the stress dependence of b-value. Attention should be paid that the estimated b-value using the power law will be very high when the differential stress is very low. Considering the sparse shallow seismicity and that the original data are mainly from depth greater than 1–2 km^14^, the lower bound depth when using the power law should also be 1–2 km.

**Figure 4:**
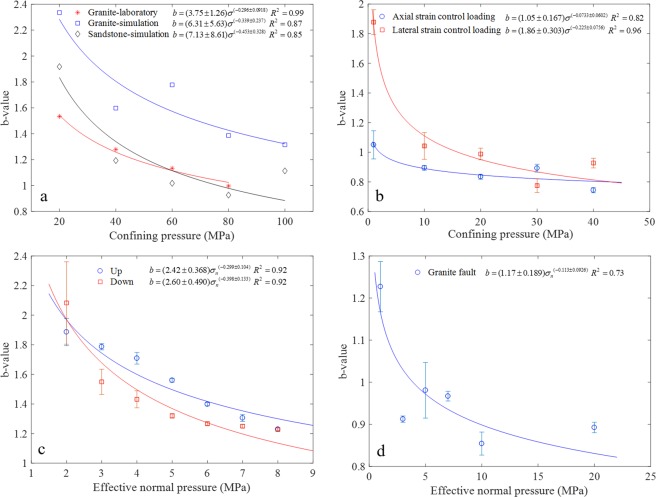
Stress dependence of acoustic emission b-value in the laboratory tests. (**a**) b-value versus confining pressure in triaxial experimental and simulation test for granite and sandstone. (**b**) b-value versus confining pressure of Hong Kong granite in the triaxial compression test loaded by axial strain and lateral strain control modes. (**c**) b-value versus normal stress in the double direct shear test with glass beads simulating the fault gouge, “up” and “down” indicate the test result during normal stress step increase and decrease respectively. (**d**) b-value versus normal stress for rough granite faults in direct shear tests.

Besides the b-value inferred from the earthquakes in favor of the power law relation, the AE b-value obtained in the laboratory earthquake simulation experiments also suggest a power law dependence on the stress. Figure 4a shows the variation of b-value for granite and sandstone with increasing confining pressure in the laboratory and numerical simulation triaxial compression tests^5^. The b-values in the uniaxial compression test are not plotted due to the different failure mechanism as compared with that obtained in the triaxial compression test. The numerical results of marble are also excluded because of its insensitivity to stress (see Fig. 15 in ref.^5^). The b-values decrease non-linearly with confining pressure, and the correlation coefficients of the fitting curves are rather high (0.85, 0.87 and 0.99 respectively) when fitting with the power law, the highest for the experimental data of granite.

We also conducted the triaxial compression test on Hong Kong granite under two different loading control modes, namely axial strain control (0.001 mm/s) and lateral strain control (0.00067 mm/s) at confining pressure from 1 to 40 MPa. The AE was monitored by 8 sensors which were attached onto the outside of the triaxial cell. The average b-value under a certain confining pressure is shown in Fig. 4b, and the error bar denotes standard deviation of the calculated b-value from the 8 sensors. The results indicate that the average b-values decrease with the power law for both loading control modes. Another group of data plotted in Fig. 4c are based on ref.^35^ after a double direct shear test under different normal stresses with glass beads simulating the fault gouge, in which periodic stick-slip under different normal stress were observed. The two data sets are obtained during normal stress increase (up) and decrease (down) process in one shear test, and the b-values calculated from the two channels of the AE system are averaged and re-plotted in Fig. 4c. The error bar denotes standard deviation of the calculated b-value from the 2 sensors. The relationship of the b-values and normal stress are well fit with the power law with high correlation coefficients (0.92). The b-value is slightly larger during the step-up phase compared to the step-down phase, which may be attributed to the gradual gouge layer thinning as the experiment proceeds^35^. Figure 4d shows the power law decrease of AE b-value with normal stress of rough and clean granite faults in the direct shear test conducted by ourselves^34^. The used granite faults are obtained by artificial splitting on cubic specimens (10 cm × 10 cm × 10 cm), and the fault surfaces are characterized by irregular and rough morphology. Four AE sensors are attached to each side of the lower part of the fault about 5 mm away from the surface. The average b-value obtained from the four sensors are used to fit the relation between the b-value and normal stress (more details about the shear tests and the calculation of b-value can be found in ref.^34^).

The above five cases, spanning from tectonic scale to laboratory scale, show that the b-value of earthquakes and AE similarly decay with the increase of stress level following the power law.

### Stress dependence of friction coefficient

Friction constitutive laws have emerged as powerful tools to describe and investigate the mechanics of earthquakes and faulting^3,45^, in which the friction coefficient is an important input variable. The friction coefficient also dictates the frictional strength at which the fault will slide^3^. Various studies have demonstrated that the friction coefficient is time- and rate-dependent^46^. The presence of water has also been believed to reduce the friction coefficients of certain minerals^36^. Here we will investigate the stress dependence of the friction coefficient based on the laboratory test data.

**Figure 5:**
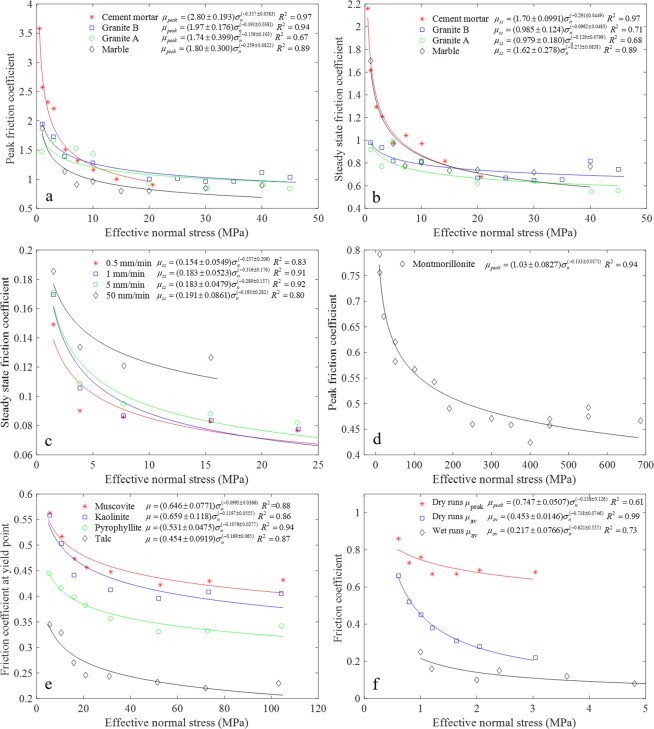
Variation of friction coefficient with stress. (**a**) Peak friction coefficient, and (**b**) Steady state friction coefficient of rough cement mortar, granite and marble faults in direct shear test. (**c**) Steady state friction coefficient of air-dried slickensided travertine joints in direct shear test. (**d**) Peak friction coefficient with increasing normal stress in the triaxial compression test with the oven-dry montmorillonite as the gouge layer between the sawcut sandstone and granite driving blocks. (**e**) Friction coefficients of different dry gouges in the triaxial compression test using steel blocks as sliders. (**f**) Friction coefficients of Wenchuan fault gouge in high-velocity rotary-shear tests with Dolerite or Gabbro as host specimen.

We conducted direct shear tests on large rough granite, marble and cement mortar faults (10 cm × 10 cm × 10 cm), which are obtained by artificial splitting. Two types of granite are used. The asperities on the joint surface are interlocked and clean without fault gouges between the interfaces. The cement mortar, which is made of a mixture of water, cement and quartz sand, is used for comparing with the results of granite and marble. Due to the limitation of the experimental condition, the three dimensional morphology of the fault surfaces are not scanned and quantified before the shear test. We chose those faults with relatively similar surface morphology to conduct the direct shear test. The applied normal stresses for granite and marble faults are in the range of 1 to 45 MPa, and 0.5 to 20 MPa for the cement mortar joints. Figure 5a,b shows the variation of peak friction coefficient *μ*_*peak*_ (defined by τ_p_/σ_n_, where τ_p_ and σ_n_ are peak shear strength and effective normal stress, respectively) and steady state friction coefficient *μ*_*ss*_ (the ratio of the residual shear strength to the effective normal stress) of the three types of rock and the cement mortar faults with the normal stresses (The shear stress-shear displacement curves can be found in ref.^47^). The maximum *μ*_*peak*_ for cement mortar fault is relatively high, i.e., 3.5 under 0.5 MPa normal stress, and then rapidly decays with increasing normal stress. We consider that the very large value of the friction coefficient under very low normal stress is attributed to those local large asperities on the joint surface. Under elevated normal stress, these asperities are likely to be sheared off and crushed between the fault surfaces, leading to the rapid decrease of the friction coefficient. The comparison of Fig. 5a,b also indicates that *μ*_*ss*_ is much lower than *μ*_*peak*_, both of which decrease following the power law.

Figure 5c shows the evolution of *μ*_*ss*_ of slickensided travertine joints with normal stress at different shear rates, and the data are from ref.^39^. Similar variation trend of *μ*_*ss*_ for the slickensided onyx marble joints were also reported. The joints surfaces were ground flat and parallel with a surface grinder and then polished using a marble diamond polishing pad. All tests were conducted on air-dried samples at room temperatures between 22 and 26 °C and 60% humidity. Similar to the decrease trend of *μ*_*ss*_ in Fig. 5b for irregular and rough fault surfaces, *μ*_*ss*_ of the polished limestone joints also decreases following the power law. However, the value of the friction coefficient for the polished joints is much less than that of the rough faults.

Natural faults may contain different types of gouges due to the frictional wear and cataclastic deformation, which will have a great influence on the frictional behavior of faults. Figure 5d shows the variation of the peak friction coefficient with normal stress in the triaxial compression test with the oven-dry montmorillonite as the gouge layer sandwiched between the sawcut sandstone and granite driving blocks (data based on ref.^36^). The friction coefficient decreases non-linearly from 0.8 to around 0.45 between 10 and 700 MPa normal stress, which can be fit by a power law with high correlation coefficient. The friction coefficients of the saturated montmorillonite at fully drained condition are much lower than those of the dry samples^36^. Increasing trend of friction coefficient is observed with the increase of confining pressure for the saturated samples^36^, indicating that the power law is more applicable to dry friction.

The influences of mineralogy and effective normal stress on frictional strength of sheet silicates were investigated using triaxial compression test, and ten different types of single-phase phyllosilicate gouges layer were sandwiched between two steel sliders^38^. The tests were conducted on oven-dried samples and under vacuum condition. The friction coefficients of four types of gouges are selected and re-plotted, as shown in Fig. 5e, and more data with similar variation trend can be found in ref.^38^. It can be seen that the power law can be used to describe the variation of friction coefficient with normal stress for all the gouge types.

To compare the friction estimated from the postseismic temperature anomaly measured across a coseismic fault zone, high-velocity friction experiments were conducted on gouge collected from outcrop and drill holes associated with the 2008 Wen Chuan Earthquake^37^. The variations of friction coefficients with different normal stresses are shown in Fig. 5f. The average friction coefficient was calculated from a combination of *μ*_*peak*_ at the peak shear strength and *μ*_*ss*_ at the steady state. The water content of the used wet gouge is 25%. The results indicate that the decrease of the friction coefficient with increasing normal stress is well fit by the power law.

The above five studies adopt different test methods, different shear rates and different materials to study the friction coefficient, i.e., direct shear test on rough granite, marble and cement mortar faults at 5 μm/s in our study, direct shear test on polished limestone joints at different shear rates^39^, triaxial compression test on montmorillonite gouge at 0.1 μm/s^36^, triaxial compression test on different phyllosilicate gouges at 0.5 μm/s^38^, and rotary-shear frictional test on Wenchuan earthquake gouges at 1.3 m/s^37^. Nonetheless, the power law decay of the friction coefficient with stress level is applicable to almost all of the studies except the saturated montmorillonite in ref.^36^.

## Discussion

The distribution of cracks (i.e., crack density) on the two sides of the faults determines the geometry and size of the fault/fracture process zone. Understanding the size and structure within fault/fracture process zone can help to evaluate fault growth models, and will also lead to a better understanding of the role of faults in fluid migration within the Earth’s crust^32^. The power law decrease of crack density with increasing distance away from the fault center is in good agreement with the theoretical prediction using an elastic-plastic fracture mechanics model considering the stress intensity factor and cohesion zone size^48^.

The proved power law between the microcrack density and fault distance is very useful. The fracture network which may control fluid migration can be estimated by measuring the microfracture density in just a few locations, rather than in a densely distributed measuring points. Besides, the obtained power law for a particular fault using several representative measuring points can be used to determine the extent of the fracture process zone.

The comparison of the fitting results in Fig. 1 indicates that the decaying rate (i.e., the exponent of the power law) of crack density is different for large scale natural faults and small scale laboratory fractures, which is around 0.2 and 0.4–0.6 respectively, suggesting that the crack density decreases faster in the laboratory fracture tests than in nature. We consider that the distribution of crack numbers around the fault may be influenced by the mineral composition of the rock and the loading condition (i.e., loading rate). Besides, the data to plot these figures are from different researchers, and the approach/criterion for identifying a microcrack in the thin section varies among different researchers, which makes the comparison of the exponents of the power law biased. These factors may be responsible for the different decay rates of natural fault and laboratory fractures.

Different scales of flaws exist in the rock mass of the Earth’s crust, from grain-scale microcracks to plate-rupturing faults. The geometry and distribution of these flaws have a great influence on the physical and mechanical behavior of geological materials under stress. It has been long recognized that the faults populations are usually scale-invariant or fractal over several orders of magnitude, which conforms to the following power law^49^:

$$N(l)=A\cdot {l}^{-D}$$

where *l* is the length of fault, *N* is the number of faults of length exceeding *l*, and *A* is a constant, *D* is scaling exponent of the length distribution (also called fractal dimension) of the system. The analysis in the present study substantiates that the induced microcracks are also fractal, which follows similar power law as the fault system.

The power law decay of earthquake/AE event number with the increase of magnitude/amplitude, and the decrease of crack number with increasing crack length both reflect the size distribution of the damage during rock fracture and fault slip process. On the other hand, the decrease of crack density, b-value and friction coefficient with increasing distance from the fault and rising stress level demonstrates the dependence of these parameters on the ambient environment. The above two processes are different in nature but similar in the formulations, which follow the power law. The number of AE events is proportional to the number of growing cracks, and the AE amplitudes are proportional to the length of crack growth increments in the rock^50^. As such, the power law decay of AE/earthquake frequency and the crack number is intimately correlated, both of which describe the size distribution of microcracks in rocks under loading.

Figure 2c indicates that the slope of the cumulative number-crack length fitting curve (similar to the b-value) does not change much at different stress levels, which is different from the decreasing trend of b-value^4^. One possible underlying reason is that the cracks with very small size are overlooked, leading to the imprecise determination of the slope of the fitting curve. Another reason is that different rock specimens have to be tested at different stress levels as compared with the AE testing in one specimen at different stages. Crack length and crack number obtained in the thin-section study after loading may subtly vary among different specimens due to the inherent heterogeneities.

For the stress dependence of b-value and friction coefficient, the power law decrease indicates that the b-value and friction coefficient are not sensitive to the change of stress in the high stress regime. The analyses of Figs 4 and 5 show that the stress at which the influence on b-value or friction coefficient become less significant varies among different studies, and is susceptible to test method, loading rate, specimen size, fault gouge, fault roughness, etc. On the other hand, the b-value was found to start to increase when the depth reaches a certain level^14^, at which the brittle-ductile transition was thought to occur. Therefore the power law decay of b-value with increasing stress is only applicable in the brittle regime, which is also supported by the simulation results of marble that the b-value starts to increase when the confining pressure is over 40 MPa at which ductile deformation occurs^5^.

**Figure 6:**
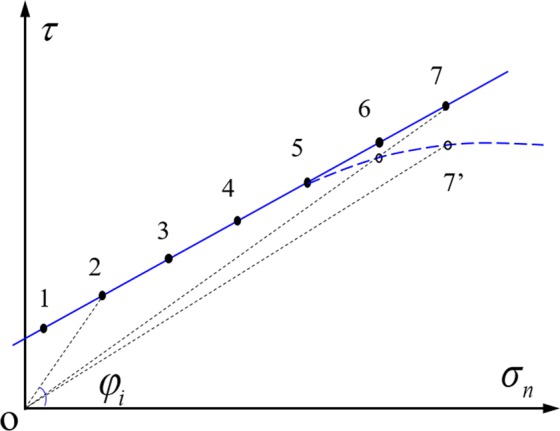
Schematic diagram of the frictional strength of faults with increasing normal stress.

The frictional strength (${\tau }_{p}$) of faults generally increases with increasing normal stress, which can be well fit with a linear relation (especially in the low normal stress range). The fitting curve may deviate from the linear trend at elevated normal stress if the rock becomes more ductile, as schematically shown in Fig. 6. Cohesion usually exists for the bare and rough fault, and is usually very small for the gouge-filled fault. Based on the definition of friction coefficient ($\mu ={\tau }_{p}$/${\sigma }_{n}=\,\tan \,{\phi }_{i}$), ${\phi }_{i}$ and *μ* will gradually decrease as normal stress increases (Fig. 6). The frictional strength (the open symbols on the dashed line) of fault typically becomes lower than the predicted value (as the solid symbols on the line) when the ductility of rock increases, which will lead to the smaller ${\phi }_{i}$ and *μ*. The friction coefficient will remain steady when the frictional strength does not vary much with stress. The above analysis can account for the decrease of friction coefficient in a power law with normal stress.

However, unlike the b-value which was found to increase with depth or stress after a certain depth or stress level is reached due to the brittle-ductile transition^14^, the friction coefficient will not increase after the decrease period because generally the fictional strength of fault will not cross the straight line in Fig. 6. This comparison indicates that the underlying mechanism for the power law decrease of b-value and friction coefficient with normal stress is different. The lower b-value at higher stress level suggests that the proportion of earthquakes with large magnitude increases due to the larger fractures with larger stress^15,51^. However, at much greater depth when the rock becomes more ductile, more earthquakes with smaller magnitude occur due to the decrease of rock strength and the ductile flow characteristics, leading to larger b-values.

The frictional strength and friction coefficient of fault or gouges vary with saturation state (or water content)^36,38^. The available strength measurements of montmorillonite gouge from the published literature were compiled and grouped into four general categories based on the water content in ref.^36^: dry, partially saturated (such as room humidity), saturated (under fully drained condition), and overpressured (saturated but undrained), in decreasing order of strength. The friction coefficient for dry montmorillonite, and for most of the partially saturated montmorillonite decreases non-linearly with increasing normal stress, which is consistent with the power law decrease of friction coefficient proposed in this study. The presence of water can significantly reduce the strength of montmorillonite gouges by the lubrication of clay platelets by thin films of adsorbed water^52^. However under high normal stress condition, the lubricating water films are increasingly expelled under fully drained condition, thus increasing the shear strength and friction coefficient. Therefore, the power law decrease of the friction coefficient is not applicable to the saturated fault gouges.

## Conclusions

Earthquake is a complicated process including both rock fracture and fault slip in micro- and macro-scale. Fault nucleation, fault growth and fault sliding are important processes during the occurrence of earthquakes. The magnitude-frequency distribution of earthquakes, the spatial distribution of hypocenters, and the frequency of aftershock occurrences are well-known scale invariance or fractal features (i.e., in power law) in the earthquakes phenomena. In this study, we compiled and analyzed a large amount of data collected from the literature and our experiments, and we find that the size distribution of microcracks during the nucleation of fracture or fault, the distribution of crack density on the two sides of small scale fracture or large scale fault, and the stress dependence of b-value and friction coefficient, which are all important parameters in the study of earthquake mechanism and prediction, follow the power law. The power law decrease of b-value and friction coefficient are controlled by different mechanisms. The findings of this study will contribute to a better understanding, modeling and prediction of earthquakes, and can also provide important clues for seismic hazard evaluation, and for the determination of fracture process zone size and the friction coefficient.
